# Tumor genomic, transcriptomic, and immune profiling characterizes differential response to first‐line platinum chemotherapy in high grade serous ovarian cancer

**DOI:** 10.1002/cam4.3831

**Published:** 2021-04-03

**Authors:** Johanne I. Weberpals, Trevor J. Pugh, Paola Marco‐Casanova, Glenwood D. Goss, Natalie Andrews Wright, Prisni Rath, Jonathon Torchia, Alexander Fortuna, Gemma N. Jones, Martine P. Roudier, Laurence Bernard, Bryan Lo, Dax Torti, Alberto Leon, Kayla Marsh, Darren Hodgson, Marc Duciaume, William J. Howat, Natalia Lukashchuk, Stanley E. Lazic, Doreen Whelan, Harmanjatinder S. Sekhon

**Affiliations:** ^1^ Department of Obstetrics and Gynecology University of Ottawa Ottawa ON Canada; ^2^ Ottawa Hospital Research Institute Ottawa ON Canada; ^3^ Department of Medical Biophysics University of Toronto Toronto ON Canada; ^4^ Princess Margaret Cancer Centre University Health Network Toronto ON Canada; ^5^ Ontario Institute for Cancer Research Toronto ON Canada; ^6^ Translational Medicine R&D Oncology AstraZeneca Cambridge UK; ^7^ Department of Medicine Division of Medical Oncology University of Ottawa Ottawa ON Canada; ^8^ Department of Pathology and Laboratory Medicine The Ottawa Hospital Ottawa ON Canada; ^9^ Quantitative Biology IMED Biotech Unit AstraZeneca Cambridge UK; ^10^ Department of Pathology and Laboratory Medicine University of Ottawa Ottawa ON Canada

**Keywords:** genomic profiling, high grade serous, immune profiling, ovarian carcinoma, platinum resistance

## Abstract

**Background:**

In high grade serous ovarian cancer (HGSOC), there is a spectrum of sensitivity to first line platinum‐based chemotherapy. This study molecularly characterizes HGSOC patients from two distinct groups of chemotherapy responders (good vs. poor).

**Methods:**

Following primary debulking surgery and intravenous carboplatin/paclitaxel, women with stage III–IV HGSOC were grouped by response. Patients in the good response (GR) and poor response (PR) groups respectively had a progression‐free intervals (PFI) of ≥12 and ≤6 months. Analysis of surgical specimens interrogated genomic and immunologic features using whole exome sequencing. RNA‐sequencing detected gene expression outliers and inference of immune infiltrate, with validation by targeted NanoString arrays. PD‐L1 expression was scored by immunohistochemistry (IHC).

**Results:**

A total of 39 patient samples were analyzed (GR = 20; PR = 19). Median PFI for GR and PR patient cohorts was 32 and 3 months, respectively. GR tumors were enriched for loss‐of‐function *BRCA2* mutations and had a significantly higher nonsynonymous mutation rate compared to PR tumors (*p* = 0.001). Samples from the PR cohort were characterized by mutations in *MGA* and *RAD51B* and trended towards a greater rate of amplification of *PIK3CA*, *MECOM*, and *ATR* in comparison to GR tumors. Gene expression analysis by NanoString correlated increased *PARP4* with PR and increased *PD*‐*L1* and *EMSY* with GR. There was greater tumor immune cell infiltration and higher immune cell PD‐L1 protein expression in the GR group.

**Conclusions:**

Our research demonstrates that tumors from HGSOC patients responding poorly to first line chemotherapy have a distinct molecular profile characterized by actionable drug targets including PARP4.

## Graphical abstract

In patients with suboptimally‐debulked, advanced high grade serous ovarian cancer (HGSOC), the spectrum of responses to first line platinum‐based chemotherapy (PtC) vary from durable to non‐existent, highlighting an unmet medical need for those with poor responses. Our study molecularly characterizes a cohort of HGSOC patients with distinct responses, either good or poor, to PtC. The good and poor responders have median progression‐free intervals of 32 and 3 months, respectively, and molecular analysis of tumors identifies differences in DNA damage response pathways and immunity, namely, tumors from poor responders to first line PtC have a distinct molecular profile characterized by actionable drug targets including PARP4.

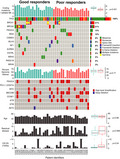

## 1 INTRODUCTION

Ovarian cancer is the leading cause of gynecological cancer mortality in North America.^1^ The primary treatment for high grade serous ovarian cancer (HGSOC), the most common subtype of this disease, consists of surgical resection and combination platinum‐based chemotherapy. Although advanced stage, poor performance status, and residual disease (RD) >1 cm after debulking surgery are strong predictors of an adverse prognosis, sensitivity to first‐line carboplatin and paclitaxel chemotherapy is a critical factor in overall outcome.^2^ However, not all patients who share adverse prognostic factors at diagnosis do poorly, suggesting there are underlying biological differences between tumors from patients at the extremes of the outcome spectrum. Patients with primary platinum‐resistance (disease‐free interval [DFI] < 6 months) have the worst prognosis with a median survival of only 9–12 months, and fewer than 15% of these patients respond to subsequent chemotherapy.^3^ Given that the biology of platinum resistance is not fully understood and the tumor characteristics predicating a poor response to treatment are largely unknown, the development of new treatment strategies has been limited, representing an impediment to new and better treatments.

Many mechanisms have been proposed to explain the variability in treatment responses to platinum‐based chemotherapy, including active efflux, enzymatic inactivation of platinum agents, and increased DNA repair capacity.^4^ Patients with platinum‐sensitive HGSOC (disease‐free interval ≥ 6 months) have seen an improvement in outcome largely attributed to the study of the DNA damage response (DDR) mechanisms as mediators of chemosensitivity.^5^ Specifically, the exploitation of homologous recombination deficiency (HRD) has led to the development and approval of PARP inhibitors in platinum‐sensitive patients with germline and somatic *BRCA1* and *BRCA2* mutations and patients with tumors displaying HRD.^6^, ^7^ The characterization of HRD beyond *BRCA1* and *BRCA2* mutation status is an active area of research and includes the study of loss of heterozygosity, epigenetic silencing of the *BRCA1* promotor, *EMSY* amplification, and the deletion of core HRD genes.^8^ Furthermore, higher PD‐L1 expression has been noted in patients with defective DDR genes including *BRCA1*,^9^ suggesting an interplay between DDR deficiency and the tumor/stromal immunophenotype, but does little to explain the >25% of patients with tumors that are resistant or refractory to platinum chemotherapy.^10^

No biomarker/signature predictive of platinum‐resistance in HGSOC has yielded clinically translatable benefit^11^ despite encouraging developments in gene expression profiling. This may be due to high levels of DNA instability in HGSOC leading to inconsistencies in reports. For instance, amplification of *CCNE1*, the gene encoding cell‐cycle checkpoint regulator Cyclin E1, has been identified as a potential marker of platinum‐resistant HGSOC^10^, ^11^ although conflicting survival data exists.^12^, ^13^ Therefore, the definition of tumor molecular characteristics associated with poor chemotherapy response is a crucial area of study with the potential to lead to early initiation of additional therapies, reduction in morbidity and cost associated with futile therapies, and informs the development of therapeutic alternatives to chemotherapy. We hypothesize that genomic and immunophenotypic features of HGSOC will characterize differential response to first‐line chemotherapy. In this study, we sought to explore these two aspects through analysis of tumors from primary debulking surgery using whole exome sequencing, whole transcriptome sequencing, targeted immuno‐ and DDR‐specific codeset profiling by NanoString gene expression panels, and immunohistochemistry (IHC).

## 2 MATERIALS AND METHODS

### 2.1 Study design and patient population

A retrospective chart review of HGSOC patients from 2004 to 2017 was conducted at The Ottawa Hospital Cancer Centre (TOHCC), Ottawa, Canada (Figure S1). Patients had advanced stage III or IV ovarian cancer, histologically confirmed HGSOC, suboptimal primary debulking with ≥1 cm RD remaining after surgery, receipt of at least four adjuvant cycles of first‐line chemotherapy with carboplatin/paclitaxel or cisplatin/paclitaxel doublet, and adequate archival tumor sample for molecular analysis. From 39 patients meeting eligibility criteria, patients were classified as having either a good response (GR, *n* = 20) or poor response (PR, *n* = 19), defined as progression‐free interval (PFI) ≥12 and ≤6 months.^12^ PFI was defined as the time between last chemotherapy treatment and disease recurrence. Assessment of response to chemotherapy and disease progression was based on RECIST v 1.1 criteria or CA‐125 progression criteria as defined by the GCIG.^13^ The study was approved by the Ottawa Health Science Network (#20150500‐01H) and University of Toronto Research Ethics Board (#34640). Consent was obtained for living patients in accordance with the declaration of Helsinki, a waiver of consent was obtained from the research ethics board for deceased patients. For enrolled patients, clinicopathologic data including PFI and overall survival (OS) were collected from the electronic medical record up to March 2018.

### 2.2 Sample preparation

Formalin‐fixed paraffin embedded (FFPE) tumor blocks were utilized for molecular analysis following quality review of a hematoxylin and eosin (H&E) slide. A reference pathologist (H.S.) identified specific tumor‐rich areas which were then macrodissected from 10‐µm tissue sections using a sterile razor blade followed by deparaffinization in 1 ml of xylene. Genomic DNA and total RNA were isolated using the Qiagen AllPrep FFPE Tissue Kit (QIAGEN) followed by fluorometric quantification using a Qubit 3.0 instrument (Life Technologies). RNA integrity was assessed using the Agilent TapeStation on a High Sensitivity RNA ScreenTape.

### 2.3 Whole exome and transcriptome sequencing

Transcriptome sequencing libraries were constructed from 200 ng of total RNA using the Illumina TruSeq Stranded Total RNA Library Prep Gold kit from which >69 million paired‐end sequencing reads were generated on the Illumina NextSeq550 platform using V2 chemistry and reagents. Exome libraries were made from 100 ng of genomic DNA using the Kapa HyperPrep Kit which were enriched using Agilent SureSelect XT Human All Exon V6+ COSMIC reagents. Exome libraries were sequenced to a mean exon coverage of 100X on the Illumina HiSeq2500 platform using V4 chemistry and reagents. All reads were processed following the GATK Best Practices framework including read alignment against the hg19 human reference using BwaMem v 0.7.12,^14^ called somatic mutations using MuTect2 v 1.1^15^ and annotated variants using Variant Effect Predictor v92.^16^ As matched normal material was lacking, likely germline variants were removed with GnomAD population frequency >0.01% in any population (r2.0.1).^17^ To control for potential FFPE‐induced sequencing artifacts, variants with allele fractions <10% were removed but retained OncoKB variants in the 5%–10% range. To assess allele‐specific copy number profiles, loss of heterozygosity, and estimates of purity and ploidy, we used CNVKIT v0.9.1^18^ using a pooled reference set of 60 peripheral blood samples from individuals unrelated to the study. To identify candidate high level amplifications and homozygous deletions, we only considered copy number variations (CNVs) with log2R > 0.7 (high level gain) and <−0.7 (deep deletions) as used by convention in cBioPortal.^19^

### 2.4 Transcriptome informatics

Total RNA data quality was assessed using FastQC and ReSeOC v 2.6.4^20^ prior to read alignment with STAR aligner v2.6.0c.^21^ BAM files were preprocessed similar to exome methods, except an additional trimming of soft‐clipped reads was performed prior to in/del realignment and base recalibration. RNA abundance was quantified with RSEM v1.3.0^22^ to generate an expression matrix. RODIC^23^ was used to identify expression outliers, ESTIMATE^24^ for immunological gene signatures/infiltrates, and ssGSEA^25^ for pathway analysis. HaplotypeCaller^26^ was used to generate variant call files (VCF) prior to Variant Effect Predictor v92^16^ analysis of annotated mutations.^27^

### 2.5 Total RNA preparation and NanoString gene expression profiling

An H&E stain was performed on one tumor section per patient, and a blinded pathologist reviewed the sections for tumor content (M.R.). The tumor component was maccrodissected from FFPE, and RNA was extracted using RNeasy FFPE Extraction kit (Qiagen) following manufacturer's instructions. RNA was quantified using Qubit 2.0 Flourometer (Life Technologies) and hybridized 150 ng of each sample with the codeset at 65°C for 21 h and kept at 4°C < 1 h before preparing cartridges. Patient samples were randomly distributed in four cartridges and included tumor reference and background controls (water). NanoString assays were performed by following the standard protocol “Setting up 12 nCounter Assays (MAN‐C0003‐03, 2008–2013)”. AZ‐designed DDR‐max codeset was used in this project. Cartridges were read immediately after being prepared, on the AZ GEN2 Digital Analyzer station with high resolution selected (3 h enhanced, 555 fields of view captured). In addition to 27 housekeeping genes, the AZ‐custom designed DDR‐max codeset includes 753 genes from the core DDR pathways and (HRD, nonhomologous end‐joining [NHEJ], mismatch repair [MMR], base excision repair [BER], nucleotide excision repair [NER], and replication stress [RS]) and previously published gene signatures predicting for response to DNA damaging chemotherapy.^28^, ^29^, ^30^, ^31^ NanoString‐designed probes were verified to ensure recognition of the canonical gene transcripts and normalized each sample with the reference samples using nSolver Analysis Software version 4.0. We then performed a housekeeping genes normalization step (assuming constant gene expression across test samples).

### 2.6 Histology and immunohistochemistry

FFPE tissues were cut into 4‐µm serial sections and mounted on charged slides. The H&E was assessed by a blinded pathologist (M.R.) for the percentage of tumor surface containing immune cells (ICs) (lymphocytes, plasma cells, and macrophages). PD‐L1 staining was conducted using Ventana PD‐L1 (SP263) IHC assay, investigational use only, according to the manufacturer's instructions on the Benchmark Ultra stainer (Ventana). PD‐L1 IHC stained slides were blindly assessed for tumor cell (TCs) PD‐L1 expression and for percentage of PD‐L1 positive ICs (over total ICs). Ataxia telangiectasia mutated (ATM) IHC staining was performed using the ab32420 antibody as previously described,^32^ and the percentage of ATM positive tumor cell nuclei was scored blindly.

### 2.7 Statistical analysis

For analyses where responder status was the predictor or independent variable, Fisher's exact test for categorical outcomes and an independent samples *t* test or a Wilcoxon rank‐sum test for continuous outcomes, significance cut‐off *p* < 0.05, were used. Associations of protein expression with clinical, pathological and molecular features, and chemotherapy response were evaluated with Fisher's exact and Wilcoxon–Mann Whitney tests, as required. To find genes associated with PFI, PFI was treated as the outcome variable in a survival model and gene expression values, as measured by the NanoString technology, were used as predictor variables in univariate analyses. PFI was modeled, adjusting for censoring, as a lognormal distribution and therefore patients with PFI values equal to zero had 0.5 added to the PFI value (all values must be >0).

## 3 RESULTS

### 3.1 Extreme response cohorts characterized by significant differences in PFI and OS

Chart review identified 39 patients that met study inclusion/exclusion criteria (Figure S1). Baseline characteristics were similar in the two extreme response groups (Table 1). There were no statistically significant differences in age, stage, *BRCA1* and *BRCA2* carrier status, residual disease, vascular invasion, or number of lines of therapy between the two groups. There was a higher median CA125 in the PR group compared to the GR group (1678 vs. 599, *p* = 0.04). At data cut‐off, the median PFI for GR and PR cohorts was significantly different at 32 and 3 months (*p* < 0.001), respectively. The median OS was 65.5 months in the GR group and 23 months in the PR group (*p* < 0.001), in support of the two groups having extremes in overall outcomes. Patients with pathogenic germline *BRCA1* and *BRCA2* mutations trended towards both a higher PFI and OS than the germline *BRCA1* and *BRCA2* negative patients.

**TABLE 1:** Study population demographic characteristics

	Total (*n* = 39)	Good responders (*n* = 20)^b^	Poor responders (*n* = 19)^b^	*p* value
Age at diagnosis, years				
Mean age at diagnosis (range) [CI]	59.4 (39−82) [56.3−62.5]	59.2 [54.0−64.4]	59.7 [55.8−63.5]	0.88
Stage				
3	36	20	16	0.11
4	3	0	3	
Ca125				
Value at diagnosis (mean)	2254	1526	2982	0.04
Value at diagnosis (median)	1085	599	1678	
BRCA germline status^a^				
Positive (Carrier)	5	4	1	0.18
Negative (WT)	31	14	17	
Unknown	3	2	1	
Residual disease				
Mean (in cm) [CI]	5.4 [4.3−6.4]	4.4 [3.1−5.8]	6.4 [4.7−8.1]	0.069
Vascular invasion				
Positive	18	11	7	0.73
Negative	16	8	8	
Not documented	5	1	4	
Total lines of chemotherapy				
Mean [CI]	2.8 [2.3−3.3]	2.4 [1.6−3.1]	3.2 [2.6−3.8]	0.09
Progression‐free interval				
Median (months)	10	32	3	<0.001
g*BRCA*+	11	50.5 (4)	0 (1)	
g*BRCA*−	6	32.5 (14)	4 (17)	
Unknown	17	25.5 (2)	3 (1)	
Overall survival				
Median (months)	41	65.5	23	<0.001
g*BRCA*+	60	76.5 (4)	37(1)	
g*BRCA*−	41	71.5 (14)	23 (17)	
Unknown	29	33.5 (2)	16 (1)	

Abbreviations: CI, 95% confidence interval; WT, wild‐type.

^a^ *BRCA1* and *BRCA2* germline mutation status as assessed by local testing.

^b^ Sample size shown within parentheses.

### 3.2 Mutations of *BRCA2* but not *BRCA1* distinguish GR and PR cohorts

**FIGURE 1:**
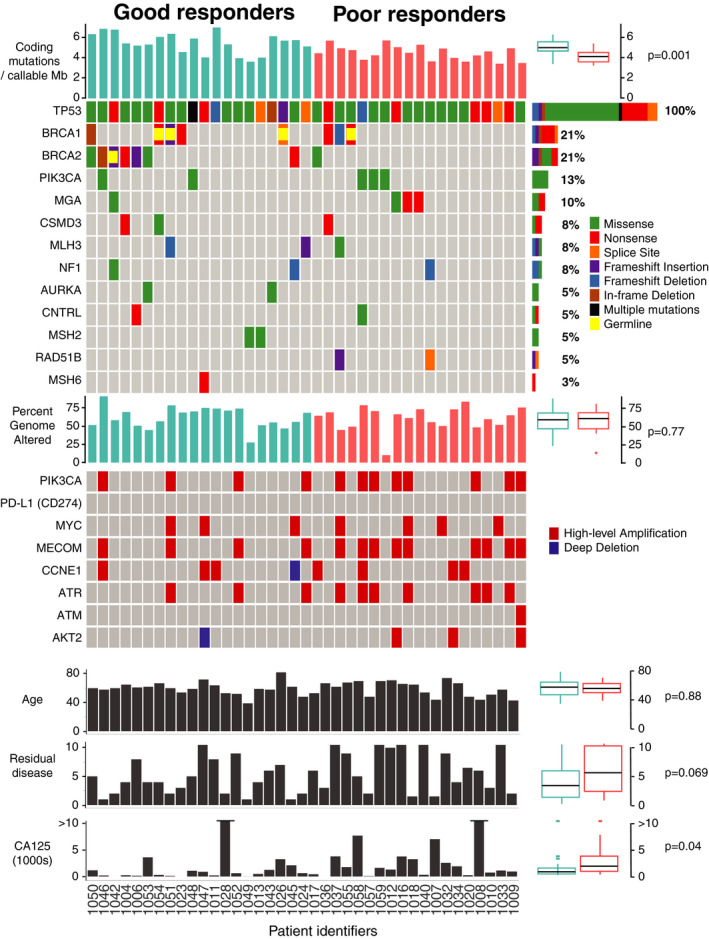
Summary of genetic alterations in the good and poor response cohorts to first line platinum doublet chemotherapy. Each column represents one patient's tumor sample. Tumor mutation burden (TMB) expressed as the number of nonsynonymous mutations per number of callable coding bases (Mb) in each sample. The value for each case is represented by a bar graph, with a summary of results to the right of the panel, expressed as a boxplot with the median bracketed by first and third quartiles and whiskers showing points within 1.5 times the interquartile range and outliers shown as individual points. *p* = 0.001 by Wilcoxon rank‐sum test. OncoPrint summary of genetic alterations (as described in adjacent legend) in the most frequently altered genes for each tumor sample. Overall frequency of alterations for each gene are listed as a percentage to the right of the panel. *BRCA1* and *BRCA2* are marked as germline mutations if germline status was reported in the clinical chart. Percent genome altered (including amplification and deletions) is shown, per subject, in a bar graph and summarized to the right of the panel as a boxplot, with *p* = 0.77 by Wilcoxon rank‐sum test. Amplifications (defined as CNVs with log2R > 0.7) and deletions (defined as CNVs with log2R < −0.7) are visually represented in an oncoprint diagram for each case. Clinical characteristics (age, residual disease, and CA125) are expressed for each patient in a bar graph, with the boxplot summary to the right of the panel, *p* values were calculated using the Wilcoxon rank‐sum test

Exome evaluation of somatic mutations in all 39 tumors revealed previously identified genes of relevance in HGSOC including *BRCA1*, *BRCA2*, and *TP53*, as well as additional oncogenic mutations in genes associated with PI3K/AKT/mTOR signaling, DDR and epigenetic regulation (Figure 1; Table S1). Comparison of tumor mutation burden found that GR tumors had significantly higher non‐synonymous mutation rate compared to the PR cohort (median 5.52 vs. 4.47 nonsynonymous mutations per callable Mb, *p* = 0.001). The most frequently mutated genes with known relevance to cancer were *TP53* (39/39 cases, 100%), *BRCA1* and *BRCA2* (15/39 including five germline, 38%), DDR genes *MLH3*, *MSH2*, *MSH6*, and *RAD51B* (8/39, 21%, none hypermutant), *PIK3CA* (5/39 all missense hotspot mutations, 13%), *MGA* (4/39, 10%), *CSMD3* (3/39, 8%), *NF1* (3/39, 2 frameshift, 8%), *AURKA* (2/39, 5%), and *CNTRL* (2/39, 5%). Of the eight somatic *BRCA2* mutations, seven were found in GR tumors (*p* = 0.044) and included an in‐frame deletion, two nonsense mutations, two frameshift insertions, and two missense mutations (one located in the helical domain). The single *BRCA2* mutation in a PR tumor was a p. Arg2108Lys missense mutation outside of a functional domain. Whereas *BRCA2* nonsense, insertion/deletion, and splice‐site loss‐of‐function (LOF) mutations were found almost exclusively in the GR group (*p* = 0.047, Fisher's exact test), LOF *BRCA1* mutations were found in both GR and PR tumors (5/20 vs. 3/19, *p* = 0.45, Fisher's exact test). LOF mutations in *MGA* (2/39) and *RAD51B* (2/39) mutations were only found in PR tumors.

Tumors were characterized by considerable aneuploidy consistent with HGSOC in both GR and PR patients, yet the percentage of genome altered by a copy number amplification or deletion was not significantly different between the two groups (median 61.32% vs. 62.32%, *p* = 0.77) (Figure 1; Table S2). An analysis of candidate genes associated with DDR and PI3K/AKT/mTOR signaling showed a trend towards greater copy number amplifications in the PR group. Specifically, PR tumors had greater number of high‐level copy number amplifications affecting *PIK3CA* (GR 4/20 vs. PR 8/19), *MECOM* (4/20 vs. 9/19), and *ATR* (3/20 vs. 7/19). *CCNE1* and *MYC* were each amplified in 18% of all cases (7/39), and this number was nearly equally distributed among PR and GR groups. In the GR group, one deep deletion was observed in *CCNE1*.

### 3.3 Differentially expressed DDR genes are correlated with chemotherapy response patterns

**FIGURE 2:**
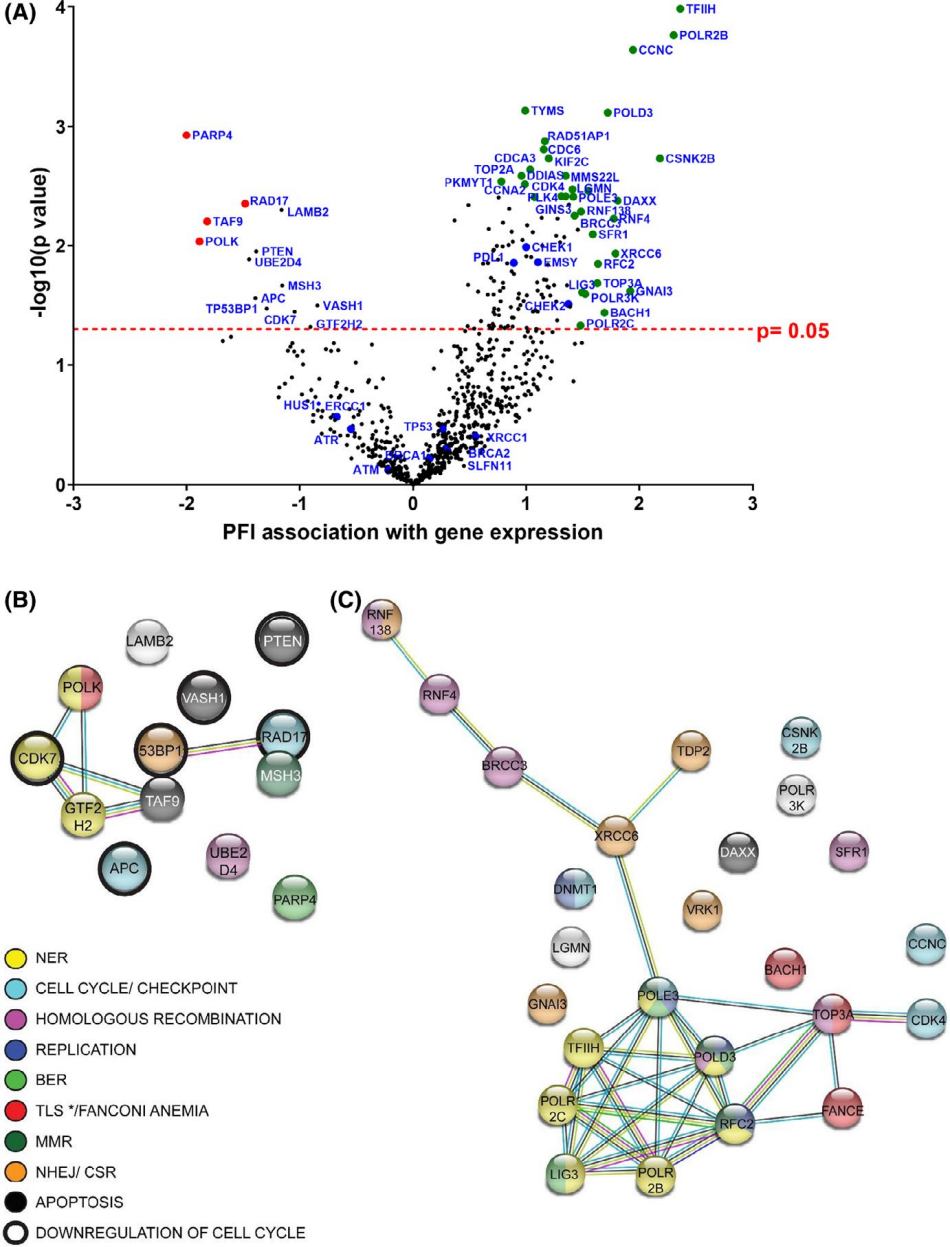
Transcriptomic analysis of tumor samples from good (GR) and poor (PR) responders using NanoString DDRmax codeset. (A) Volcano plot showing correlation of gene expression with patient PFI (X axis, greater value correlates with longer PFI). Y axis represents the level of confidence of the statistical analysis for each gene (*p* value 0.05 is depicted by a dashed line and was used as a cut‐off value). Green and red dots: genes best correlated to long and short PFI, and with lowest *p* value (list expanded in Table S3), blue dots: DDR genes closely examined. Protein association networks of top genes the expression of which correlates best with shorter PFI (B) and longest PFI (C) (left and right genes on volcano plot, respectively), with a *p* value < 0.05. Color code refers to curated KEGG pathways, where * denotes that TLS is specific to the short PFI network. Pathways were generated with String software. Red color refers to Translesion synthesis in panel (B) and Fanconi Anemia pathway in panel (C). BER, base excision repair; MMR, mismatch repair; NER, nucleotide excision repair; NHEJ/CSR, nonhomologous end joining/class‐switch recombination; TLS, translesion synthesis

To test whether there was correlation between clinical response and expression of DDR genes, a specific DDR panel codeset for NanoString analysis was applied, specifically suited to provide high‐quality results in RNA extracted from FFPE tissue. The analysis by NanoString nCounter of 753 DDR genes of the 39 HGSOC tissues (20 GR, 19 PR) showed that 127 genes were expressed at significantly higher levels in GR patients, whereas 13 genes were significantly more expressed in PR patients. *TFIIH*, *POLR2B*, *CCNC*, *LIG3*, *POLD3*, *RFC2*, *POLE3* genes were amongst the most highly expressed in GR samples. In contrast, PR tumors had elevated levels of *PARP4*, *POLK*, *CDK7*, *RAD17* and *TP53BP1* (Figure 2A; Table S3). Analysis of protein association networks carried out for the 127 genes or selected subsets (top 20% of genes with highest correlation to PFI (Figure 2C; Figure S2A), or top 20% of genes with the lowest *p* value) showed that GR tumors were enriched for expression of genes involved in DNA replication/cell cycle, BER, NER, and HRD pathways (measured by lowest false discovery rate [FDR]) (Figure S2C). In contrast, the PR group showed an enrichment of genes associated with negative regulation of cell cycle (i.e., *PTEN*, *APC*, and *VASH1*). The PR tumors also showed an enrichment of genes involved in NER (Figure 2B; Figure S2B).

**FIGURE 3:**
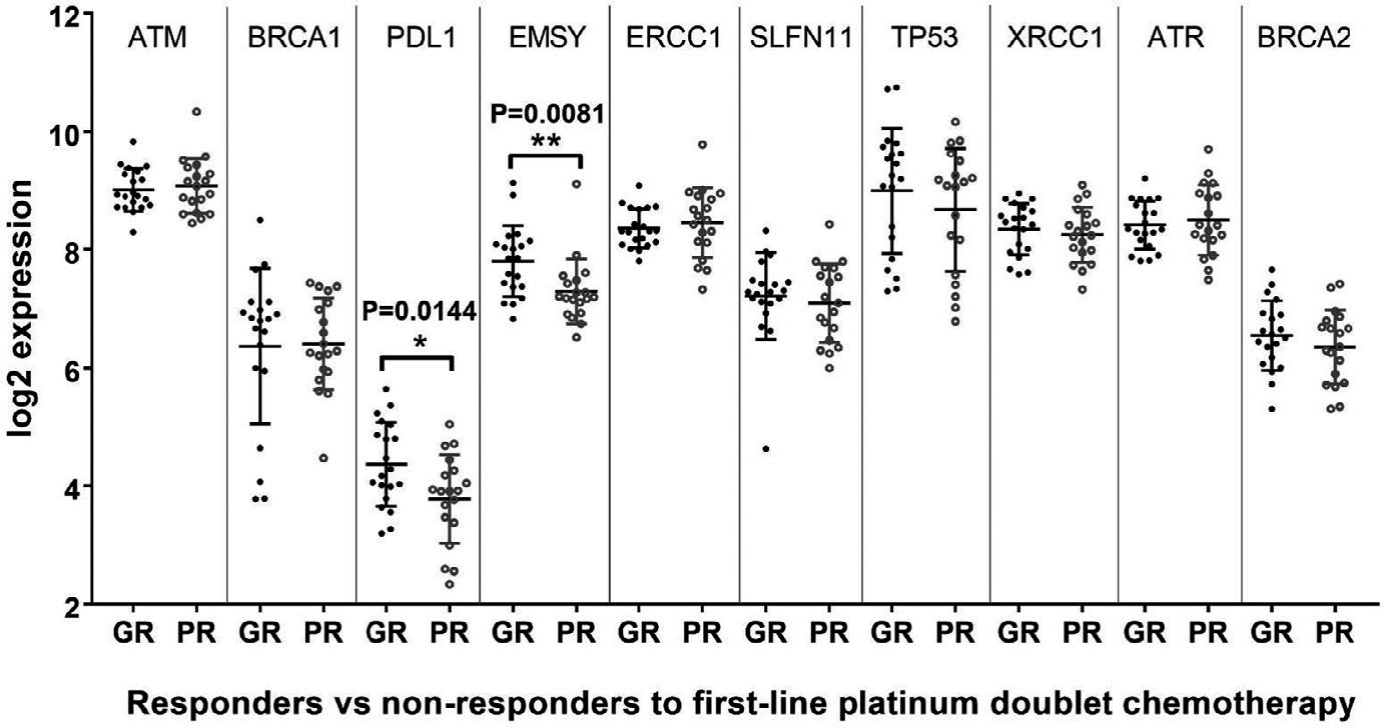
Transcriptomic analysis of tumor samples from good (GR) and poor (PR) responders showing selected NanoString gene expression data. Gene expression (log2 expression) values for selected genes shown with mean ± SEM for each response group, where GR, good response; PR, poor response. Data analyzed with R‐limma package (version 3.30.13), multiple hypotheses not tested

The expression levels of selected genes known to be relevant in DDR pathways were examined by NanoString nCounter (Figure 3). Expression of two genes implicated in the response to DNA damaging chemotherapy were significantly higher in the GR group, *EMSY* (*p* = 0.008), a repressor of *BRCA2* transactivation; and programmed death ligand 1 (PD‐L1) (*CD274*) (*p* = 0.014), an immune inhibitory receptor ligand frequently used as a biomarker for immune checkpoint inhibition (Figure 3). There were no amplifications in *EMSY* or *CD274* in any case tested (*CD274* shown in Figure 1). Although the *ATR* gene was more frequently amplified in the PR group (Figure 1), this did not translate to a significant difference in *ATR* mRNA expression between GR and PR groups when pooled (Figure 3). Furthermore, although *ATR* gene expression (mRNA z‐Scores) from RNA‐seq trended towards correlation with gene copy number, NanoString analysis showed no statistically significant difference in mRNA log2 gene expression when amplified cases were examined compared to all others, independently of platinum‐response (Figure S3A,B). ATM expression was also evaluated and although all samples were ATM‐expressing, neither RNA expression nor protein expression (IHC) was differential between response groups (Figure 3; Figure S4A). In addition, no notable DNA alterations to *ATM* were found (data not shown). Furthermore, no significant differences in mRNA expression were found between the GR and PR groups for the DDR genes: *BRCA1*, *BRCA2*, *ERCC1*, *SLFN11*, *TP53*, and *XRCC1* (Figure 3).

**FIGURE 4:**
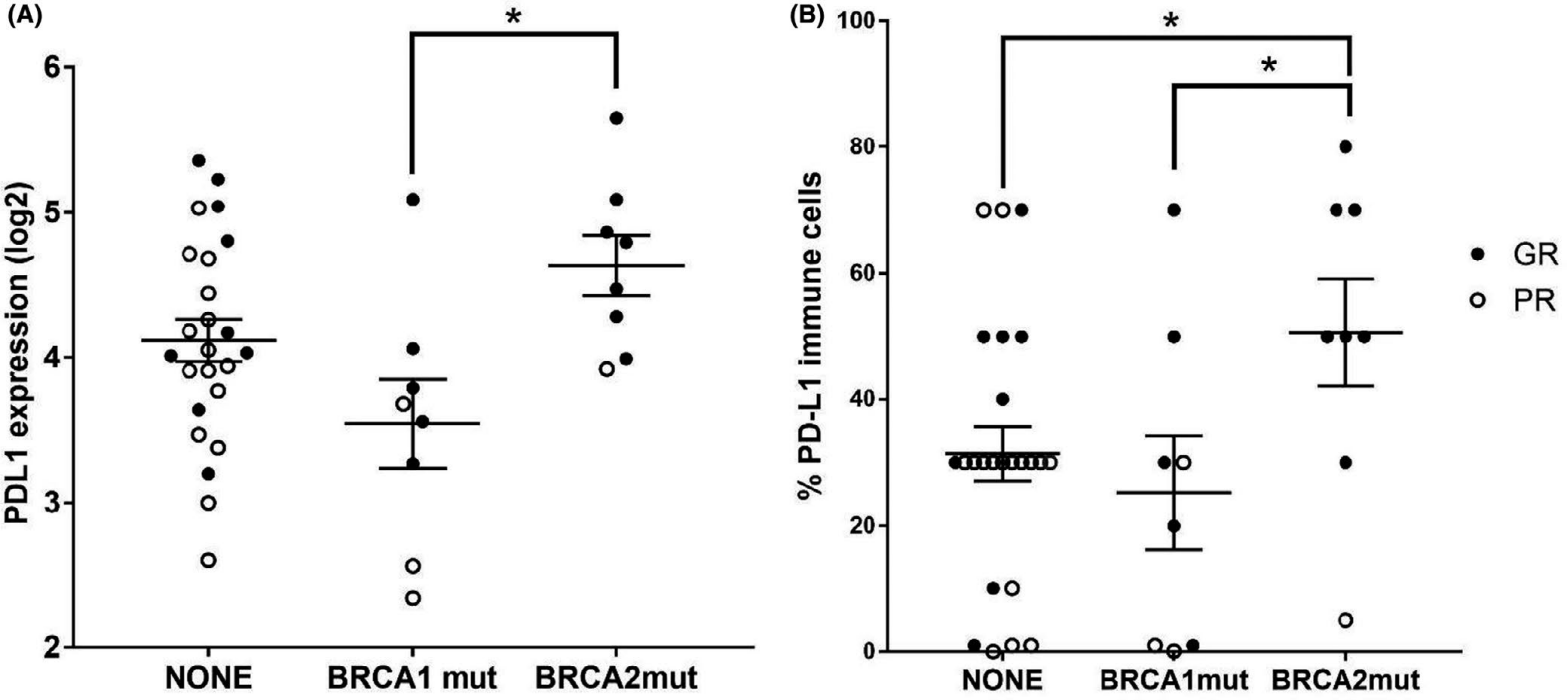
Transcriptomic and immunohistochemical (IHC) analysis of tumor samples from good (GR) and poor (PR) responders establishes a link between *BRCA* mutation status and PD‐L1 expression in tumors and tumor associated immune cells. (A) PD‐L1 mRNA expression in tumors from both response groups classified by *BRCA1* and *BRCA2* mutation status. Data shown as mean ± SEM (* indicates *p* = 0.013). (B) Percentage of PD‐L1 protein expression in tumor immune cells, assessed by IHC, within tumors from both response groups, based on *BRCA1* and *BRCA2* mutation status. Data shown as mean ± SEM (* and ** indicate *p* = 0.029 and *p* = 0.01, respectively)

Increased PD‐L1 mRNA levels in the GR group correlated with *BRCA2* mutation status (*p* = 0.013) but not with *BRCA1* mutations (Figure 4A) and PD‐L1 protein expression in tumor immune cells was significantly higher in those patients with mutations in *BRCA2* (*p* = 0.029) (Figure 4B). *EMSY* expression did not significantly correlate with *BRCA* status (Figure S4B).

### 3.4 Increased intratumoral inflammation and immune cell PD‐L1 expression correlates with chemotherapy response

**FIGURE 5:**
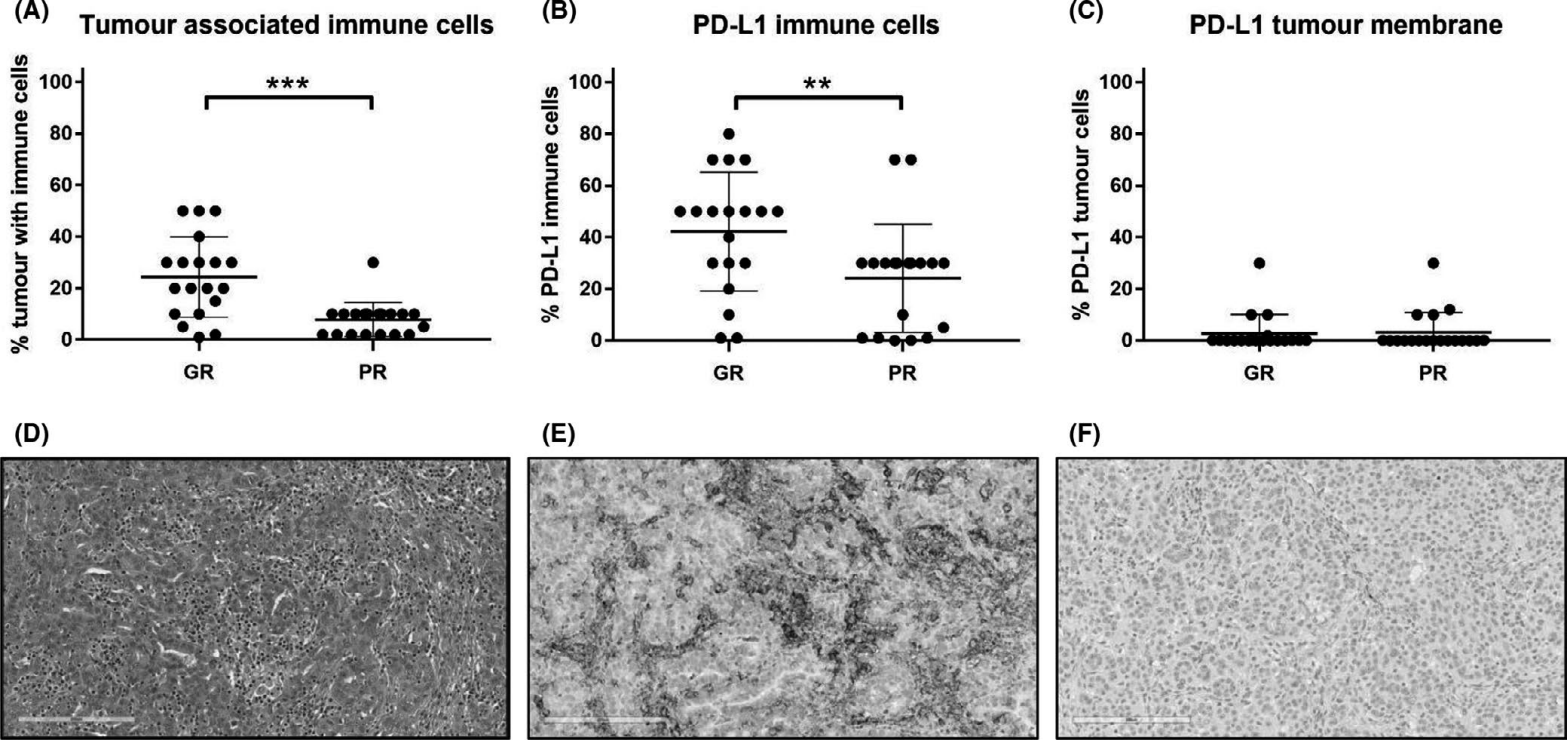
Immunohistochemical evaluation of patient tumor specimens: immune cell infiltration and PD‐L1 expression on the surface of tumor cells (TC) and immune cells (IC). (A) Percentage of tumor associated immune cells in responders compared to nonresponders to first‐line platinum doublet chemotherapy, using H&E ***p < 0.001. (B) Amongst immune cells (ICs), percentage of PD‐L1 positive ICs were significantly higher in responders as compared to nonresponders. (PD‐L1 positivity defined as 25% membrane positivity in the absence of an established definition for HGSOC) **p < 0.01. (C) Percentage of PD‐L1 positive tumor cells in both response cohorts. No difference between GR and PR groups. (D) Representative H&E of a good responder tumor with dense inflammation (immune cells are morphologically lymphocytes, plasma cells and macrophages), 20×. (E) Same tumor as in (D) stained with SP263 showing patchy infiltration of tumor stroma by PD‐L1 positive immune cells. PD‐L1 (SP263) 20×. (F) Poor responder tumortumor with absence of positive PD‐L1 tumor associated immune cells. PD‐L1 (SP263) 20× (PD‐L1 antibody: SP263, IUO Ventana Benchmark assay)

Based on the positive association of PD‐L1 gene expression with favorable chemotherapy response, PD‐L1 IHC was conducted to confirm increased protein expression and delineate tissue expression patterns. Intratumoral inflammation estimated by H&E was significantly higher in the GR group at 24% (±16% SD) than in the PR group at 8% (±7% SD) (Figure 5A,D). In addition, the mean percentage of PD‐L1 expressing immune cells by IHC was higher in GR tumors than in PR tumors at 42% (±23% SD) and 24% (±21% SD), respectively (*p* < 0.001 Mann–Whitney) (Figure 5B,E,F), based on the number of positive cells. PD‐L1 protein expression was low in tumor cells, and there was no difference in PD‐L1 expression between the two groups (Figure 5C). Immune cell infiltration was either patchy in the stroma (Figure 5E), observed at the invasive margin (Figure S5A) or diffuse in between tumor cells (Figure S5B).

To assess whether these patterns may be associated with specific immune infiltrates, RNA‐seq of 29 tumors (Table S5) was performed to assess total infiltration (ESTIMATE algorithm) and infiltration of specific immune cell types (CIBERSORT). Overall, we did not find a significant difference in aggregate immune or stromal scores between the GR and PR groups (Figure S6), nor were there significant differences using a focused analysis of 22 immune cell signatures (Figure S7). Ultimately, larger cohorts or high‐resolution single cell expression analysis may yield insight into these patterns.

## 4 DISCUSSION

### 4.1 Patients with similar clinical and demographic characteristics exhibit distinct survival outcomes in HGSOC

Platinum resistance poses a great clinical challenge in the treatment of HGSOC as up to 30% of patients develop recurrent disease within 6 months of first line treatment^10^ and virtually all patients eventually fail platinum therapy. In the treatment of patients with suboptimal debulking, the addition of bevacizumab to systemic chemotherapy is a therapeutic option; however this strategy lacks a predictive biomarker and has no overall survival benefit when used in the front‐line setting.^33^ The options for treatment of poor prognosis patients in the first‐line setting are limited, stressing the need to explore novel therapies. In this study, we compare the molecular profile of tumors from patients undergoing primary debulking surgery, who have had good and poor responses to first‐line platinum‐based chemotherapy.

The GR and PR cohorts in the present study are noteworthy for their extreme differences in median PFI and OS (32 months vs. 3 months, and 65.5 months vs. 23 months, respectively). In suboptimally debulked stage III, HGSOC patients treated with platinum doublet chemotherapy, the previously reported PFI and OS were 14.1–16.9 and 35–45.1 months, respectively,^2^ suggesting that our cohort truly represents two distinct outcome groups. Given our limited cohort of patient specimens with variable responses, it is noteworthy that our molecular findings corroborate results from large‐scale genomic characterisation efforts in stage II–IV HGSOC tumors. Thus, suggesting that many molecular features are in fact characteristics of the disease, rather than markers of response. An example of this is the ubiquitous mutation rate of *TP53*, which is 100% in our tumor cohort.^34^, ^35^

### 4.2 Alterations in genes responsible for DNA repair mechanisms characterize GR and PR groups

The *BRCA1*and *BRCA2* mutation rate of 38% (15/39) in the present study is higher than the The Cancer Genome Atlas (TCGA) reported rate of 22%,^35^ although the selection of good responders likely enriches our *BRCA* mutant cohort. Our finding that *BRCA2* mutations were significantly more frequent in the GR group is in accordance with evidence that mutations in *BRCA1* and especially *BRCA2*, confer chemo‐sensitivity in ovarian cancer.^5^ Retrospective studies have correlated *BRCA1* and *BRCA2* mutations, higher tumor mutation burden and infiltration by T cells with improved survival in HGSOC,^36^, ^37^ and *BRCA2* mutations were found to be enriched among long‐term responders to PARP inhibition.^38^

Aside from expected mutations in *BRCA1* and *BRCA2* and *TP53*, mutated tumor suppressor genes are uncommon in HGSOC.^35^ Rather, HGSOC is characterized by marked genomic instability, with frequent DNA gains and losses and up to 50% of tumors demonstrating HR deficiency.^35^ Terms like BRCAness and HRD characterize the myriad genes that can produce a defective DNA repair phenotype and influence sensitivity to agents such as chemotherapy and PARPi. One gene belonging to this group is *EMSY*, a repressor of *BRCA2* transactivation that localizes to sites of DNA repair and the amplification of which has been associated with an adverse prognosis.^39^ Amplification of *EMSY*, and consequent suppression of *BRCA2* transcriptional activity, has been proposed as an additional mechanism of defective DNA double‐strand break (DSB) repair in HGSOC.^40^ TCGA data suggest that the impact of *BRCA*ness on the chemo‐responsiveness of a tumor is dependent on the mechanism by which *BRCA* is silenced (e.g., mutation vs. epigenetic silencing do not carry the same predictive value).^35^ In this study, there were no amplifications or mutations in *EMSY* at the DNA level, but *EMSY* mRNA levels were significantly increased within the GR group, supporting a role for *EMSY* upregulation as a marker of chemosensitivity.

Ataxia telangiectasia mutated (ATM) and Rad3‐related protein (ATR) are core players in the DDR pathway, with central roles coordinating response to DSBs and replication stress (RS).^41^ RS represents the initial insult from stalled or collapsed DNA replication forks during oncogenesis, which places ATR in a pivotal position to confront growing genomic instability within a developing cancer. Therefore, this provides rationale for ATR's role in inherent chemotherapy resistance, as it stabilizes stalled replication forks and allows DSB repair.^42^ Although *ATR* was not mutated in our cohort and its expression was not significantly different in GR and PR samples, we report *ATR* amplification, as determined by exome sequencing, at a higher frequency (26%, 10/39 cases) than in the TCGA datasets (8%).^19^, ^43^ When correlated with response, the divide grew, with *ATR* amplification noted in 15% of GR and in 37% of PR, but this was not statistically significant in this relatively small cohort.

### 4.3 Differential genetic alterations in MYC/PI3K pathways between response groups

Amplification of Chromosome 3q (Chr3q) has been frequently described in up to 50% of HGSOCs.^44^, ^45^ Our study noted amplifications in several genes of interest located on Chr3q including *ATR*, *MECOM*, and *PIK3CA*. That *PIK3CA*, which is commonly amplified in HGSOC (18%),^35^ was amplified in our study predominantly in the PR group (42% of cases), is noteworthy. The amplification rate of *MECOM* and *CCNE1* in our study cohort was consistent with TCGA data.^35^ Our study also found various genetic alterations detected in *MGA* that were concentrated in the PR group. MGA, a transcription factor which interacts with c‐MYC, is a putative tumor suppressor gene. Inactivating mutations in MGA may contribute to solid tumor development and have been detected in colorectal cancer, adenocarcinomas of the lung and small‐cell lung cancers.^46^, ^47^

### 4.4 NanoString gene expression analysis highlights differential expression of DDR gene between response groups

The broad screen of mRNA expression data using the NanoString DDRmax codeset showed association between longer survival and high expression of genes involved in DNA replication, NER, BER, and HR repair pathways.

The expression of genes such as *TFIIH*, *POLR2B*, *POLR2C*, *CCNC*, *RFC2*, *LIG3*, and *POLD3*, involved in NER and transcription, correlated with PFI (Figure 2A). We also observed an enrichment of NER genes in the PR group, which is in agreement with previous data relating high expression of NER genes to platinum resistance in ovarian carcinoma, as platinum‐DNA adducts are resolved by NER mechanisms.^48^ These data are also consistent with other reports showing that low expression of *POLD3* is a marker of poor prognosis.^49^ Select HR genes were also overexpressed in patients with long PFI, as it is the case of *MMS22L*, *RAD51AP1*, *RAD54B*, and *RAD54L*. Noticeably, the overexpression of genes involved in DNA replication (*CDC6*, *CDT1*, *MCM2*, *MCM3*, *MCM5*, *MCM10*, *CDK2*, *CCNA2*) in patients with long PFI links enhanced replication potential, on which platinum relies to exert DNA damage, with GR (Figure 2A; Figure S2A; Table S3). Conversely, genes highly expressed in tumors from individuals with short PFI were enriched in processes that negatively regulate cell cycle progression. This is in agreement with previous reports^50^ suggesting that a slower proliferation rate would enable cells to repair damaged DNA. In the PR group, genes *PARP4* and *RAD17* were more highly expressed (Figure 2A). RAD17 recruits the 9‐1‐1 complex in response to RS to activate ATR, hence its higher expression in patients with lower PFI could contribute to their resistance to platinum therapy. *ATR* and *HUS1* (which encodes one of the components of the 9‐1‐1 complex) exhibited the same trend but lacked significance (below cut‐off in Figure 2A). Therefore, more efforts into the study of DNA replication genes as potential biomarkers predicting the efficiency of platinum‐based therapy are warranted.

Several reports link *PARP4* overexpression with multidrug resistance genes such as *MVP*, and, in particular, it was shown by IHC methods that higher levels of PARP4 correlate with higher grade ovarian cancer.^51^, ^52^ Furthermore, high expression of *PARP4* has been reported in breast cancer with poor outcomes.^53^ Several PARP inhibitors have already been approved for cancer treatment and, in light of our results and those of previous reports, PARP4 emerges as a candidate actionable target for platinum‐resistant HGSOC.

### 4.5 Higher immune context in the GR cohort linked to *BRCA2* mutations

Our finding that patients with good chemotherapy response have a higher tumor mutation burden is consistent with other studies.^37^, ^54^, ^55^ Evidence supports that the predicted neo‐antigen load is higher in *BRCA1*‐ and *BRCA2*‐mutant and HR‐deficient cancers,^54^ owing to the theory that impaired DDR leads to genetic alterations and putative neo‐antigens, thus favoring recognition by the immune system.

The connection between *BRCA1* and *BRCA2* gene mutations and PD‐L1 expression has been previously described in HGSOC, with some discordance in the literature. This study makes the important observation that tumors from patients with *BRCA2* mutations had increased PD‐L1 mRNA expression and a higher percentage of PD‐L1 protein expression in tumor‐associated ICs compared to patients with *BRCA1* mutations. This is in agreement with two studies reporting that PD‐L1 expression correlated with *BRCA1* and *BRCA2* mutation status,^54^, ^56^ but in contrast to another, which reported no association with *BRCA1* or *BRCA2* mutation, or somatic mutation load, using TGCA data.^57^ PD‐L1 expression and CD8+ TILs are associated with favorable prognosis in HGSOC and this was found regardless of the extent of RD following cytoreduction, receipt of standard treatment and germline *BRCA1* status.^57^, ^58^, ^59^ Taken together, our results, support the existing body of literature characterizing *BRCA1* and *BRCA2* mutations as predictive of chemo‐response and potentially associated with high tumor immune cell infiltration and PD‐L1 IHC expression. However, this observation does not necessarily correlate with long‐term survival benefit.^56^

In primary ovarian tumors, PD‐L1 expression has been predominantly reported on immune cells, rather than on tumor cells^57^, ^60^ and our results corroborate a low tumor cell expression level of PD‐L1. Although our analyses examined all immune cells, increased PD‐L1 IC expression has been primarily reported in CD65+ tumor‐associated macrophages. Expression of PD‐L1 in HGSOC has been described as focal and patchy in one large study and the frequency of CD8+ TIL positivity (Grade 2/3 and Stage I–III) is reported to be around 95%, with 57.4% of cases being PD‐L1 positive and 37.4% being PD‐L1 negative.^57^ This high positivity rate is greater than we observed but the discrepancy may be due to methodology differences and the immune cell types examined. Agents that recruit CD8+ T cells to the tumor milieu such as immunostimulants (i.e., cytokines) and DNA damaging agents that promote immunogenic cell death (such as chemotherapy), may alter the tumor microenvironment to favorably influence therapeutic responses and therefore new combination therapies are an area of clinical interest.

### 4.6 Future directions

In this analysis, as no matched normal control samples were available, it was not possible to definitively identify *BRCA1* and *BRCA2* mutations as somatic or germline in all patients. Results of germline testing were not available on all patients at the time of data collection. As we did not have matched normal controls, all genomic findings underwent stringent filtering to ensure false positive signals were sufficiently reduced. The differential amplification in *PIK3CA*, *MECOM*, and *ATR* while interesting signals require validation using an orthogonal method and a larger cohort given the high degree of aneuploidy found in ovarian carcinoma.

Despite these limitations, our gene expression data supports a clear role for DNA repair genes in both the favorable and poor response groups. Our work suggests interplay between DDR pathways and the tumor immune microenvironment in shaping platinum response. Durable remissions were associated with a high tumor mutation burden, evidence of DNA repair disruption (via *BRCA2* mutations and *EMSY* overexpression), and tumor infiltration of PD‐L1 expressing immune cells. Platinum resistant tumors were characterized by increased expression of PARP4, NER pathway genes, and lower expression of DNA replication genes as compared to platinum sensitive tumors. These findings may be applied to larger studies to advance therapeutic options for patients experiencing inherent platinum resistance.

## CONFLICT OF INTEREST

GG has acted as principal investigator on an AstraZeneca‐sponsored clinical trial, outside the submitted work. GG and JW have participated in AstraZeneca advisory boards, outside the submitted work. GG has received travel grants from AstraZeneca, outside the submitted work. GG and JW have received honoraria from AstraZeneca, outside the submitted work. MR, GNJ, SEL, PMC, DH, and NL are/were employees of AstraZeneca, and MR, GNJ, PMC, DH, and NL own AstraZeneca stock. The other authors declare no potential conflicts of interest relevant to this publication.

## AUTHOR CONTRIBUTIONS

Johanne I Weberpals: study conception, study design, data acquisition, data analysis, data interpretation, manuscript writing, manuscript review, and approval; Trevor J. Pugh: study design, data acquisition, data analysis, data interpretation, manuscript writing, manuscript review, and approval; Paola Marco‐Casanova: study design, data acquisition, data analysis, data interpretation, manuscript writing, manuscript review, and approval; Glenwood D. Goss: study conception, study design, data analysis, data interpretation, manuscript review, and approval; Natalie Andrews Wright: data acquisition, data analysis, data interpretation, manuscript writing, manuscript review, and approval; Prisni Rath: data acquisition, data analysis, data interpretation, manuscript review, and approval; Jonathon Torchia: data acquisition, data analysis, data interpretation, manuscript review, and approval; Alexander Fortuna: data acquisition, data analysis, data interpretation, manuscript review, and approval; Gemma N Jones: data acquisition, data analysis, data interpretation, manuscript review, and approval; Martine P Roudier: data acquisition, data analysis, data interpretation, manuscript review, and approval; Laurence Bernard: data analysis, data interpretation, manuscript review, and approval; Bryan Lo: data analysis, data interpretation, manuscript review, and approval; Dax Torti: data acquisition, data analysis, data interpretation, manuscript review, and approval; Alberto Leon: data acquisition, data analysis, data interpretation, manuscript review, and approval; Kayla Marsh: data acquisition, manuscript review, and approval; Darren Hodgson: study conception, study design, data interpretation, manuscript review, and approval; Marc Duciaume: data acquisition, data analysis, manuscript review, and approval; William J Howat: data acquisition, manuscript review, and approval; Natalia Lukashchuk: data analysis, data interpretation, manuscript review, and approval; Stanley E. Lazic: data analysis, data interpretation, manuscript review, and approval; Doreen Whelan: data acquisition, data analysis, manuscript review, and approval; Harmanjatinder S. Sekhon: study design, data acquisition, data analysis, data interpretation, manuscript review, and approval.

## Supporting information

Fig S1‐S7

Table S1

Table S2

Table S3

## Data Availability

Some of the data that supports the findings of this study are available in the supporting information of this article (i.e., deidentified data), and the remainder of the data that support the findings of this study are available on request from the corresponding author, these are not publicly available due to privacy or ethical restrictions (i.e., associations of clinical data and molecular data).
