# Supplementary material for: Tumor genomic, transcriptomic, and immune profiling characterizes differential response to first‐line platinum chemotherapy in high grade serous ovarian cancer
Source: Cancer Med. 2021 Apr 3;10(9):3045–58. doi: 10.1002/cam4.3831 (PMC8085970; doi:10.1002/cam4.3831)
Supplement: Supplementary file 1 — Fig S1‐S7 [file CAM4-10-3045-s004.pdf]

A

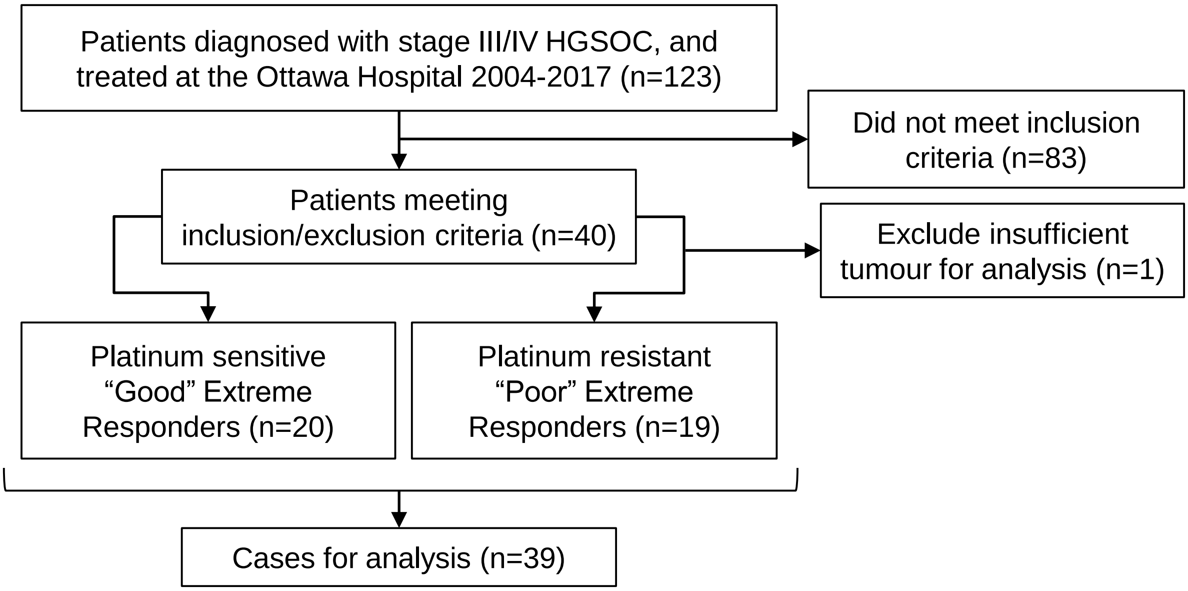

B

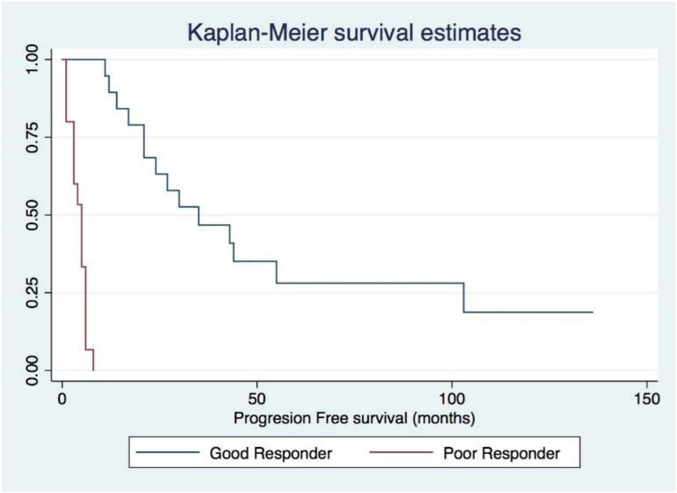

C

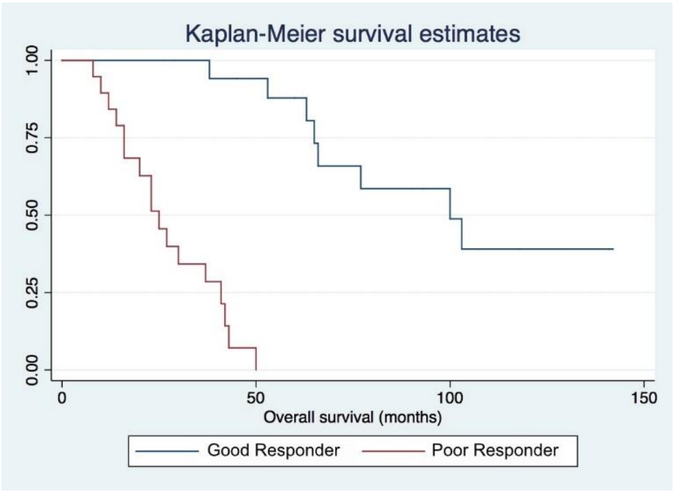

Supplemental Figure 1

A

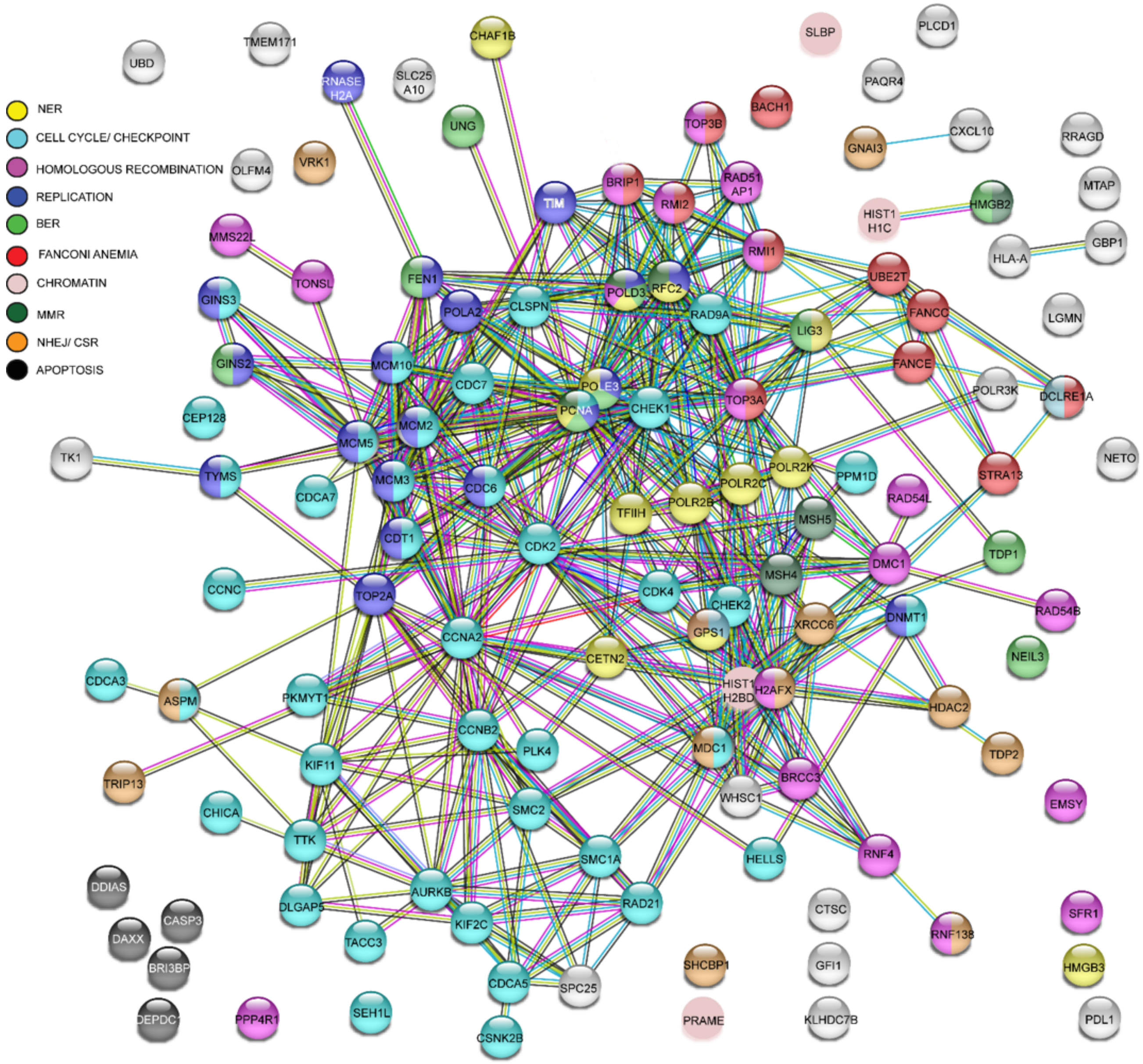

Pathway ID	Type	Description	False discovery rate
hsa04110	KEGG	cell cycle	4.15E-15
hsa03030	KEGG	DNA replication	1.96E-11
hsa03460	KEGG	Fanconi Anemia (FA)	7.30E-09
hsa03410	KEGG	Base excision repair (BER)	2.07E-07
hsa03420	KEGG	Nucleotide excision repair (NER)	1.72E-05
hsa3440	KEGG	Homologous recombination (HR)	2.40E-04
hsa03430	KEGG	Mismatch repair (MMR)	5.20E-03
hsa03450	KEGG	NHEJ	3.08E-02

B

Pathway ID	Type	Description	False discovery rate
GO:0045786	Biological process (GO)	Negative regulation of cell cycle	9.43E-05
hsa05200	KEGG	Pathways in cancer	4.60E-04
hsa03420	KEGG	Nucleotide excision repair (NER)	3.40E-03

C

Pathway ID	Type	Description	False discovery rate
hsa03420	KEGG	Nucleotide excision repair (NER)	1.69E-05
hsa03410	KEGG	Base excision repair (BER)	2.10E-04
hsa03030	KEGG	DNA replication	2.10E-04
hsa03020	KEGG	RNA polymerase	2.10E-04
hsa3440	KEGG	Homologous recombination (HR)	2.40E-04
hsa03430	KEGG	Mismatch repair (MMR)	4.10E-03

Pathway ID	Type	Description	False discovery rate
hsa03420	KEGG	Nucleotide excision repair (NER)	1.40E-04
hsa03410	KEGG	Base excision repair (BER)	1.00E-03
hsa03030	KEGG	DNA replication	1.00E-03
hsa03020	KEGG	RNA polymerase	1.00E-03
hsa3440	KEGG	Homologous recombination (HR)	1.10E-03
hsa04110	KEGG	Cell cycle	1.30E-03
hsa03460	KEGG	Fanconi Anemia pathway (FA)	1.70E-03
hsa03430	KEGG	Mismatch repair (MMR)	9.40E-03

Supplemental Figure 2

A

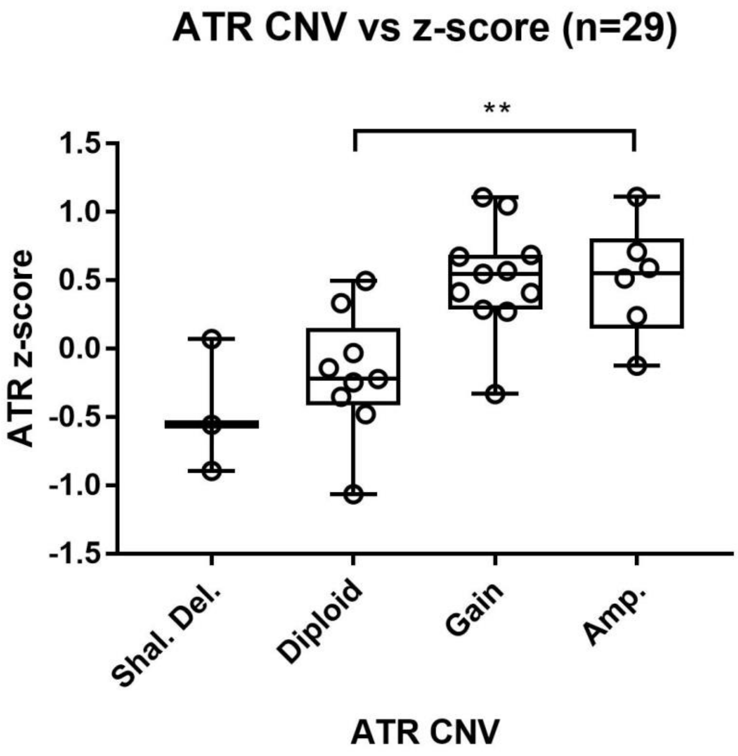

P= 0.0076
Two-tailed Mann-Whitney

B

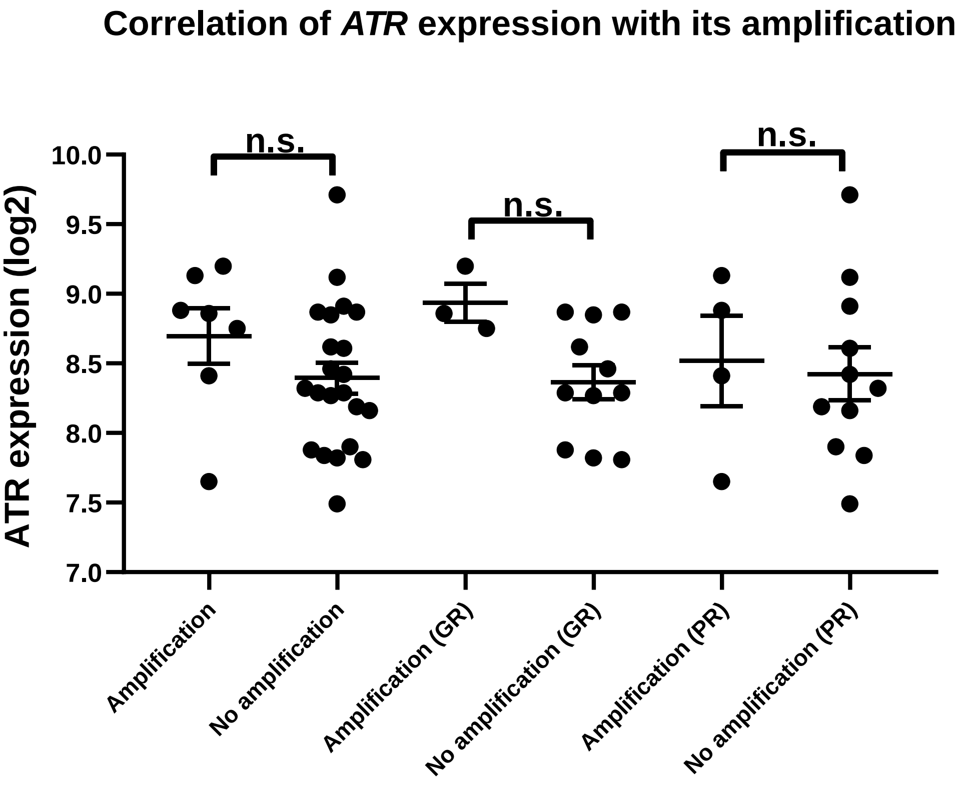

Total -> p = 0.1318

GR group -> p = 0.797

PR group -> p = 0.7531

Supplemental Figure 3

A

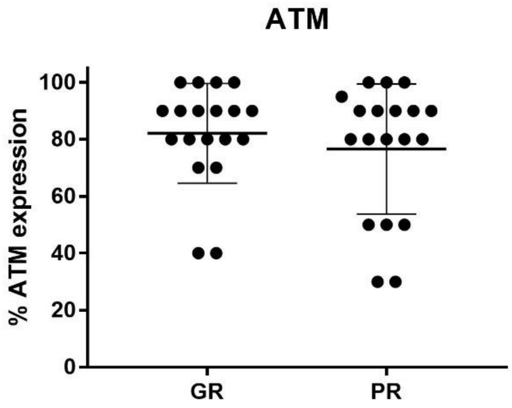

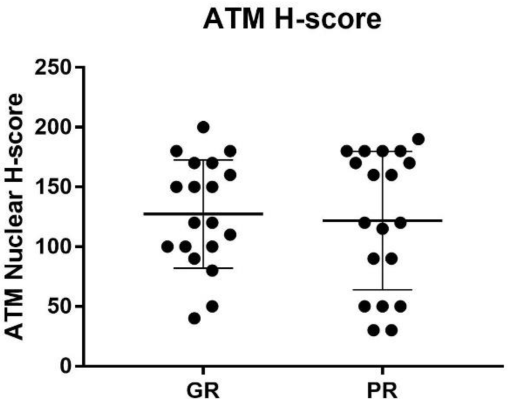

ATM, 80%, Responder

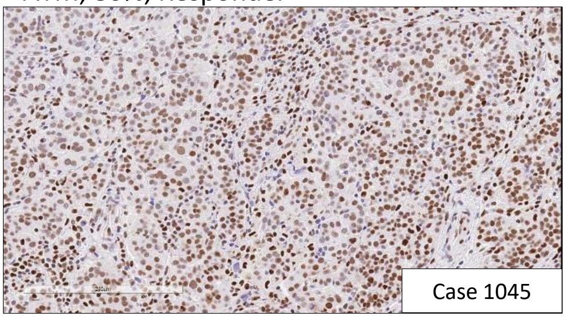

ATM, 100%, Non-responder

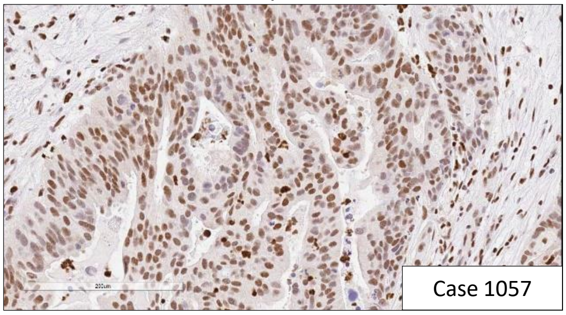

B

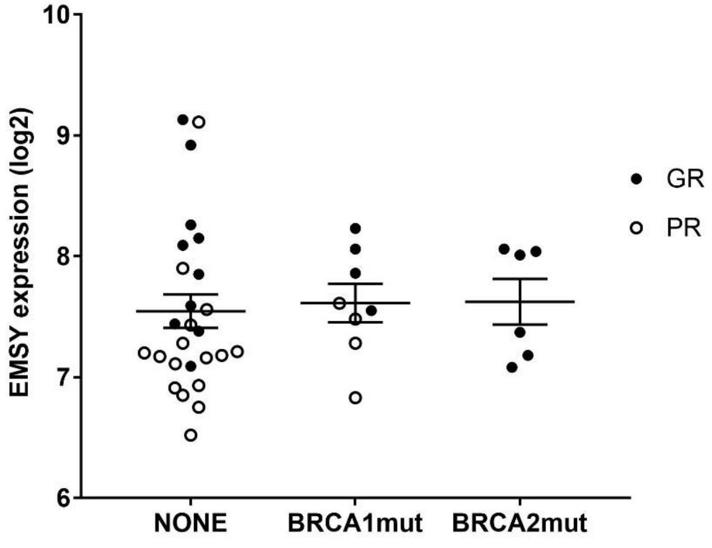

Supplemental Figure 4

A B

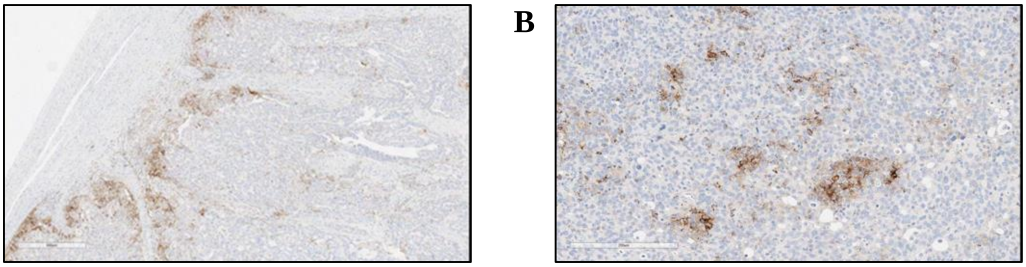

Supplemental Figure 5

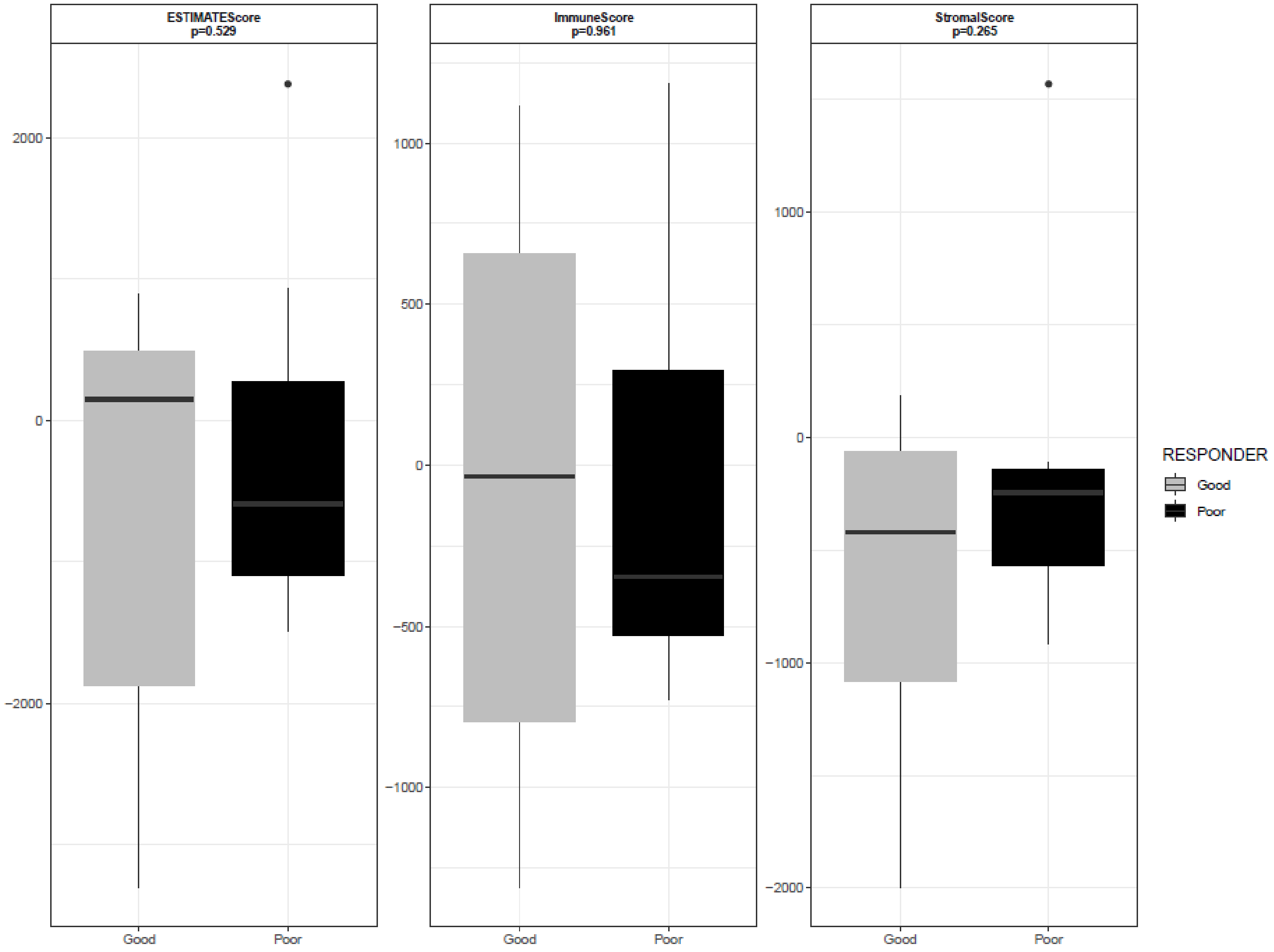

Supplemental Figure 6

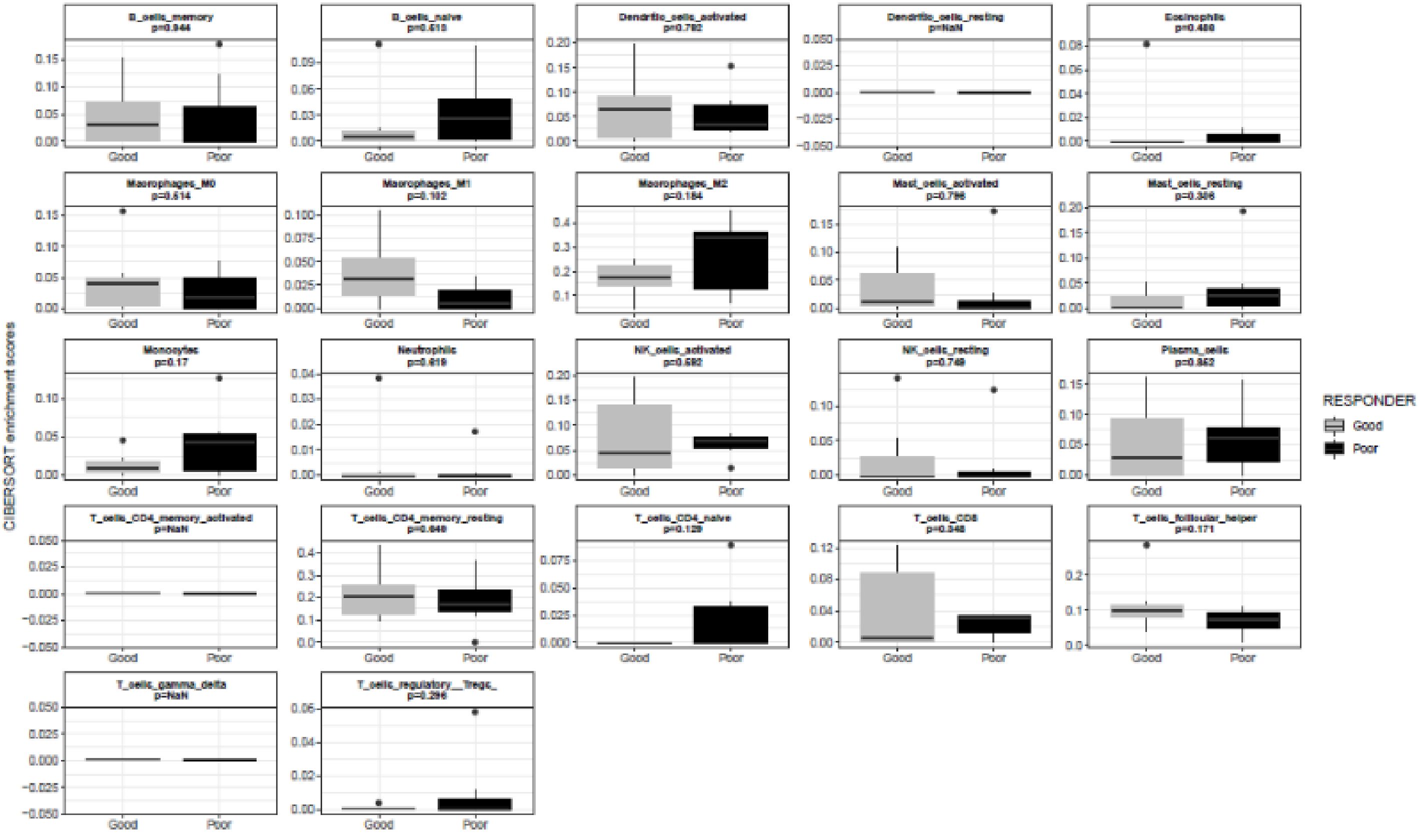

Supplemental Figure 7
